# Nothing Great Comes Without Its Risks: A Rare Case of Pembrolizumab-Induced Hypophysitis

**DOI:** 10.1210/jcemcr/luad135

**Published:** 2023-12-19

**Authors:** Ashen Fernando, Aastha Mittal, Rashid Cheema

**Affiliations:** Department of Internal Medicine, Geisinger Medical Center, Danville, PA, USA 17822; Department of Internal Medicine, Geisinger Medical Center, Danville, PA, USA 17822; Department of Endocrinology, Geisinger Medical Center, Danville, PA, USA 17822

**Keywords:** hypophysitis, pembrolizumab, immune check point inhibitors, immune-related adverse effects

## Abstract

Pembrolizumab is an immune checkpoint inhibitor that targets the programmed cell death protein 1 and enhances immune activity against cancer cells. This has emerged as a powerful tool in the treatment of cancer in patients with severe metastatic disease. Despite this, immune checkpoint inhibitors are associated with many immune-related adverse effects. Reported endocrinopathies include thyroid dysfunction, insulin-deficient diabetes mellitus, primary adrenal insufficiency, and hypophysitis. Hypophysitis is more commonly associated with cytotoxic T-lymphocyte associated antigen 4 inhibitors like ipilimumab and rarely with pembrolizumab. A high clinical suspicion is needed to pursue a diagnosis of immune checkpoint inhibitorinduced hypophysitis, and prompt diagnosis is of immense importance due to the potentially life-threatening nature of endocrinopathies. We present a case of a 64-year-old Caucasian male individual undergoing treatment with pembrolizumab for undifferentiated lung carcinoma who subsequently developed hypophysitis.

## Introduction

Immune checkpoints are proteins expressed on the surface of immune cells and are crucial for maintaining self-tolerance and preventing autoimmunity. Immune checkpoint inhibitors (ICPis) are antibodies that antagonize certain immune checkpoints leading to enhanced antigen-specific T-cell activation and antitumor activity. This ingenious mechanism of action recently emerged as a powerful tool in the treatment of cancer and has been shown to improve outcomes and disease-free survival [1]. However, its use has been jeopardized by the development of various immune-related adverse effects (IRAEs). Endocrinopathies are among the more common IRAEs related to ICPi therapy. These include thyroid dysfunction, insulin-deficient diabetes mellitus, primary adrenal insufficiency, and hypophysitis. Cytotoxic T-lymphocyte associated antigen 4 (CTLA-4) and programmed cell death protein 1 (PD-1) are 2 immune checkpoints that have been studied and have demonstrated effective and durable antitumor activity in patients with certain solid and hematological tumors [2]. ICPi-induced hypophysitis most commonly affects the anterior pituitary gland and is particularly associated with CTLA-4 agents and is less common with PD-1 inhibitors such as pembrolizumab. To our knowledge, only 28 patients have been diagnosed with hypophysitis following pembrolizumab [3, 4]. We present a case of hypophysitis diagnosed 8 weeks following initiation of pembrolizumab for non-small cell lung carcinoma.

## Case Presentation

A 64-year-old Caucasian male individual was evaluated in the endocrinology clinic for abnormal thyroid laboratory results. He had a medical history of thoracic SMARCA4- deficient undifferentiated lung carcinoma with metastasis to the right cerebellar and left occipital lobes. He was treated with 3 fractions of 2400 cGy radiation therapy. He subsequently was started on pembrolizumab and trastuzumab therapy. A complete blood count, comprehensive metabolic panel, and thyroid function tests obtained prior to initiation of treatment were all unremarkable. He received 3 cycles of pembrolizumab and trastuzumab. The treatment was well tolerated as only mild fatigue and rash were reported. Following his third cycle, he presented with fatigue and cold intolerance. On further review of the systems, he was found to have constipation, dry skin, weight gain, poor libido, erectile dysfunction, hot flashes, diaphoresis, and flushing. He denied headaches, visual impairment, diplopia, lightheadedness, changes in appetite, polyuria, polydipsia, nocturia, or dry mouth.

## Diagnostic Assessment

**Figure 1.**
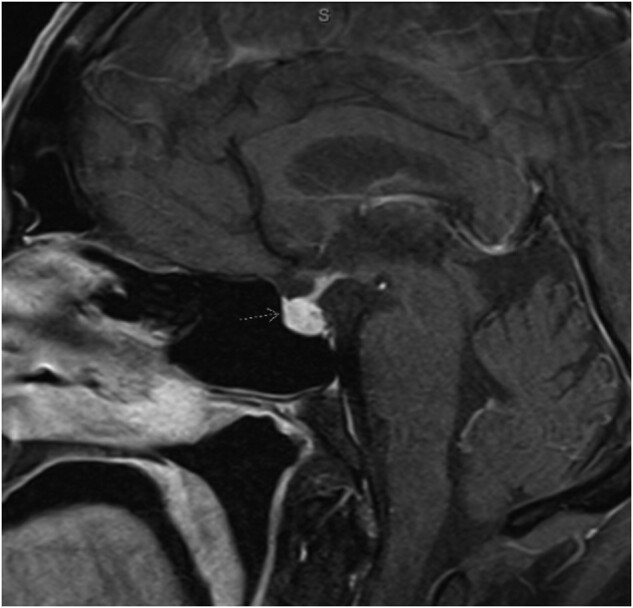
MRI while on pembrolizumab, showing new enlargement and homogeneous enhancement of the pituitary gland and infundibulum with convex superior margin.

His vital signs were within normal limits. On physical examination, he was a well-appearing individual, with an intact field of vision and a nonpalpable thyroid gland. Initial laboratory studies showed a sodium of 143 mEq/L [143 mmol/L] (135-146 mEq/L; 135-146 mmol/L), potassium of 4.8 mEq/L [4.8 mmol/L] (3.5-5.1 mEq/L; 3.5-5.1 mmol/L), thyroid stimulating hormone (TSH) of 0.09 uIU/mL [0.63 pmol/L] (0.27-4.20 uIU/mL; 1.88-29.2 pmol/L), thyroxine (T4) of 0.8 ng/dL [0.02 nmol/L] (0.9-1.7 ng/dL; 0.03-0.05 nmol/L) and T3 of 2.2 pg/mL [3.38 pmol/L] (2.5-4.3 pg/mL; 3.84-6.61 pmol/L), consistent with central hypothyroidism. Morning serum cortisol was 11.1 ug/dL [306 nmol/L] (2.5-19.5 ug/dL; 69-538 nmol/L) and adrenocorticotropic hormone (ACTH) was 26.4 pg/mL [5.81 pmol/L] (7.2-63.3 pg/mL; 1.59-13.9 pmol/L). The decision was made to proceed with a cosyntropin stimulation test. His serum cortisol prior to administration of cosyntropin was 21.9 ug/dL [604 nmol/L] and serum cortisol 30 minutes following administration 26.1 ug/dL [720 nmol/L]. He was also noted to have low total testosterone of 19.5 ng/dL [0.68 nmol/L] (193-740 ng/dL; 6.69-25.7 nmol/L) and free testosterone 4.5 pg/mL [16.5 pmol/L] (35-130 pg/mL; 128-477 pmol/L) with inappropriately normal follicular stimulating hormone (FSH) and luteinizing hormone (LH) consistent with central hypogonadotropic hypogonadism (Table 1). Magnetic resonance imaging (MRI) of the brain showed new enlargement and homogeneous enhancement of the pituitary gland and infundibulum with convex superior margin but no mass effect on the optic chiasm suggestive of hypophysitis (Fig. 1).

**Table 1.** Laboratory values

	Baseline lab values	Posttreatment with pembrolizumab	On levothyroxine and hydrocortisone
Sodium (135-146 mEq/L; 135-146 mmol/L)	143 mEq/L (143 mmol/L)	142 mEq/L (142 mmol/L)	139 mEq/L (139 mmol/L)
Potassium (3.5-5.1 mEq/L; 3.5-5.1 mmol/L)	4.8 mEq/L (4.8 mmol/L)	4.4 mEq/L (4.4 mmol/L)	3.8 mEq/L (3.8 mmol/L)
Creatinine (0.6-1.2 mg/dL; 0.05-0.11 mmol/L)	1.2 mg/dL (0.11 mmol/L)	1.2 mg/dL (0.11 mmol/L)	1.3 mg/dL (0.11 mmol/L)
Glucose (70-120 mg/dL; 3.89-6.66 mmol/L)	113 mg/dL (6.27 mmol/L)	121 mg/dL (6.71 mmol/L)	111 mg/dL (6.16 mmol/L)
TSH (0.27-4.20 uIU/mL; 1.88-29.2 pmol/L)	1.66 uIU/mL (11.6 pmol/L)	0.09 uIU/mL (0.63 pmol/L)	1.86 uIU/mL (13 pmol/L)
Free T4 (0.9-1.7 ng/dL; 0.03-0.05 nmol/L)	1.1 ng/dL (0.03 nmol/L)	0.8 ng/dL (0.02 nmol/L)	1.5 ng/dL (0.04 nmol/L)
Free T3 (2.5-4.3 pg/mL; 3.84-6.61 pmol/L)	3.5 pg/mL (5.38 pmol/L)	2.2 pg/mL (3.38 pmol/L)	3.0 pg/mL (4.61 pmol/L)
FSH (1.5-12.4 mIU/mL; 1.5-12.4 IU/L)		3.3 mIU/mL (3.3 IU/L)	6.2 mIU/mL (6.2 IU/L)
LH (1.7-8.6 mIU/mL; 1.7-8.6 IU/L)		2.2 mIU/mL (2.2 IU/L)	5.4 mIU/mL (5.4 IU/L)
Total testosterone (193-740 ng/dL; 6.69-25.7 nmol/L)		19.5 ng/dL (0.68 nmol/L)	229.8 ng/dL (7.97 nmol/L)
Free testosterone (35-130 pg/mL; 128-477 pmol/L)		4.5 pg/mL (16.5 pmol/L)	51.1 pg/mL (187.6 pmol/L)
Sex hormone binding globulin (1.14-8.65 ug/dL; 12-91 nmol/L)		1.42 ug/mL (15 nmol/L)	1.99 ug/mL (21 nmol/L)
Morning serum cortisol (2.5-19.5 ug/dL; 69-538 nmol/L)		11.1 ug/dL (306 nnmol/L)	0.4 ug/dL (11 nmol/L)
ACTH (7.2-63.3 pg/mL; 1.59-13.9 pmol/L)		26.4 pg/mL (5.81 pmol/L)	< 3.0 pg/mL (<0.6 pmol/L)
Cosyntropin- 0 minutes		21.9 ug/dL (604 nmol/L)	
Cosyntropin- 30 minutes		26.1 ug/dL (720 nmol/L)	
Prolactin (4-15.2 ng/mL; 4-15.2 ug/L)		10.3 ng/mL (10.3 ug/L)	
IGF-1 (41-279 ng/mL; 5.3-36.5 nmol/L)		109 ng/mL (14.3 nmol/L)	
Copeptin (<63.6 pg/mL; < 14 pmol/L)		35.4 pg/mL (7.8 pmol/L)	

Abbreviations: ACTH, adrenocorticotropic hormone; FSH, follicle-stimulating hormone; IGF-1, insulin-like growth factor 1; LH, luteinizing hormone; T3, triiodothyronine; T4, thyroxine; TSH, thyrotropin (thyroid stimulating hormone).

## Treatment

The patient was started on levothyroxine 50 mcg daily. He also began taking hydrocortisone 20 mg in the morning and 10 mg in the afternoon. He underwent an additional cycle of chemotherapy without pembrolizumab due to biochemical and radiologic evidence of hypophysitis. Although there was laboratory evidence of central hypogonadotropic hypogonadism, he was not started on testosterone replacement at the initial visit, with plans to reassess pituitary gonadal axis.

## Outcome and Follow-Up

**Figure 2.**
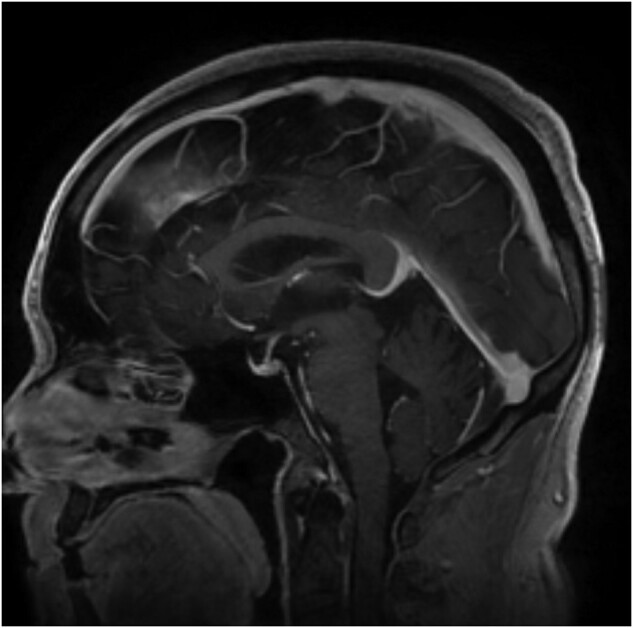
MRI following withdrawal of pembrolizumab and initiation of steroid therapy.

Repeat MRI showed radiological improvement in hypophysitis (Fig. 2). Upon reassessment of the hypothalamic pituitary axis, he was found to have a low morning serum cortisol level of 0.4 ug/dL (2.5-19.5 ug/dL). However, his serum total and free testosterone levels showed improvement to normal levels. His TSH and T4 levels were within normal levels while on hormone replacement (Table 1). He was eventually restarted on pembrolizumab and continued on levothyroxine and hydrocortisone.

## Discussion

Hypophysitis is characterized by chronic inflammatory infiltration of the pituitary gland, which over time causes the pituitary gland to become replaced by fibrotic tissue. This results in both disruption of its architecture and function. Hypophysitis can be classified based on either etiology or histopathological findings. Primary hypophysitis is autoimmune in nature, occurring without a clear cause [5, 6]. This type of hypophysitis originates and is limited to the pituitary gland. Secondary hypophysitis occurs when the inflammation is a result of a clearly identified systemic disorder, such as sarcoidosis, granulomatosis with polyangiitis, hemochromatosis, amyloidosis, infection, or medications. With regards to histopathology, hypophysitis can be classified as lymphocytic, granulomatous, plasmatic, xanthomatous, or necrotizing [2, 5, 6].

In recent times, ICPis have emerged as having significant potential in treating cancer by promoting intrinsic antitumor activity. Nevertheless, several IRAEs have been reported in the literature. Although the frequency of endocrine dysfunction is not well established, hypophysitis is considered a rare occurrence that is only diagnosed when it is severe (grade 3 or 4) [5]. Hypophysitis was initially reported to only occur with CTL-4 inhibitors, but more recently PD-1 inhibitors have also been shown to cause hypophysitis. Compared to the incidence of hypophysitis with the use of CTL-4 inhibitors, the incidence is considerably lower with PD-1 inhibitors (reported to be as low as 0.5%) like pembrolizumab [5]. This observed difference could be explained by the presence of CTL-4 and the absence of PD-1 ligands in normal pituitary tissue [5]. In addition, the presence of certain genetic mutations involving various cell signaling pathways such as PTEN, BRAF, and EGFR influences the expression of PD-1 antigens in hypophysis tissue, which may contribute to an alternate mechanism for IRAEs [5]. Most ICPi-related endocrinopathies occur within 6 to 12 weeks of initiating therapy, but there are several cases of developing IRAEs several months to years after initiation [2, 5]. Involvement of the anterior pituitary gland is more common than the posterior pituitary gland [2]. The most common hormone deficiencies seen in ICPi-induced hypophysitis are deficiencies in TSH, FSH, LH, and ACTH. Interestingly, central hypothyroidism and hypogonadotropic hypogonadism can be transient, and these hormonal axes may recover spontaneously [2, 5]. Dysfunction of the pituitary adrenal axis in most cases seems to persist [2, 4, 5]. A normal cosyntropin stimulation test as observed in our patient cannot definitively exclude adrenal insufficiency [2]. This is because a normal rise in cortisol can still be seen earlier in the disease process, as it takes time for the adrenal glands to atrophy in response to diminished ACTH secretion. This is further supported by the fact that our patient later developed adrenal insufficiency.

Patients with anterior hypophysitis usually present with nonspecific symptoms and most commonly include headache and fatigue [3, 5]. This can often lead to delayed clinical recognition leading to dire consequences, predominantly mediated by central adrenal insufficiency. The diagnosis of ICPi-related hypophysitis is based on a combination of biochemical and radiographic findings. MRI is the imaging modality of choice, showing diffuse enlargement of the pituitary gland and diffuse enlargement and irregular thickening of the infundibulum. Mass effect on the optic chiasm is rare [2]. However, approximately 23% of cases may have normal MRI despite clinical evidence of hypophysitis [5, 6].

Treatment of ICPi-induced hypophysitis involves hormone replacement. Withdrawal of ICPis is only recommended in grades 2 to 4 but could be continued alongside hormone replacement therapy in grade 1 hypophysitis [5]. In our case, immunotherapy was initially held by the oncologist; however, it was later resumed while the patient was receiving adequate hormone replacement therapy. Although the benefit of glucocorticoids in patients with grades 1 to 2 hypophysitis remains unclear, low-dose corticosteroids may be used to mitigate pituitary inflammation or as hormone replacement therapy [5, 7]. The European Society of Medical Oncology recommends the use of oral steroids at 1 to 2 mg/kg in grade 2 toxicity, whereas in grade 1 only the respective hormone replacement is recommended [5]. Although administration of high-dose glucocorticoids is considered the cornerstone in the management of patients with critical illness (grades 3-4) related either to hypophysitis or hypopituitarism, its use remains controversial as it has not shown to significantly alter the time for resolution of pituitary enlargement [5]. According to the European Society of Medical Oncology guidelines, an intravenous dose of 1 to 2 mg/kg methylprednisolone for 3 to 5 days followed by 1 to 2 mg/kg of oral prednisone with gradual tapering in 4 weeks is recommended for grade 3 to 4 hypophysitis [5]. The decision to treat our patient with steroid replacement even with a normal cosyntropin stimulation test was made based on his clinical assessment, concern for relative adrenal insufficiency with his stress cortisol level being < 13 ug/dL [359 nmol/L], and risk of progression to adrenal crisis in the setting of ICPi use.

Some degree of pituitary function may recover 4 to 12 weeks following withdrawal of immunotherapy. Given the increasing use of ICPis in oncologic practice and the potentially life-threatening nature of endocrinopathies if not promptly recognized, it is critical for the treating clinicians to consider IRAEs when patients present with nonspecific complaints. It is also important for endocrinologists and oncologists to be aware of the clinical manifestations, diagnosis, and management of ICPi-related endocrinopathies.

## Learning Points

- Although rare, PD-1 inhibitors like pembrolizumab are associated with hypophysitis.
- Hypophysitis should be considered in patients receiving immunotherapy who present with unexplained headaches and fatigue.
- Diagnosis of ICPi-related hypophysitis should be based on a combination of biochemical and radiographic findings in addition to a high clinical suspicion.
- Prompt treatment should be initiated with hormone replacement and steroid therapy depending on the grade of hypophysitis.

## Contributors

All authors made individual contributions to authorship. R.C. was involved in the diagnosis and management of this patient and reviewing the final manuscript. A.F. and A.M. were involved with writing and editing the manuscript for submission. All authors reviewed and approved the final draft.

## Data Availability

Original data generated and analyzed during this study are included in this published article.

## Abbreviations

ACTH: adrenocorticotropic hormone
FSH: follicle-stimulating hormone
ICPi: immune checkpoint inhibitor
LH: luteinizing hormone
MRI: magnetic resonance imaging
PD-1: programmed cell death protein 1
T3: triiodothyronine
T4: thyroxine
TSH: thyrotropin (thyroid stimulating hormone)
